# Prevalence, antimicrobial resistance, and genotyping of Shiga toxin-producing *Escherichia coli* in foods of cattle origin, diarrheic cattle, and diarrheic humans in Egypt

**DOI:** 10.1186/s13099-021-00402-y

**Published:** 2021-02-05

**Authors:** Walid Elmonir, Samar Shalaan, Amin Tahoun, Samy F. Mahmoud, Etab M. Abo Remela, Radwa Eissa, Hanem El-Sharkawy, Mustafa Shukry, Rasha N. Zahran

**Affiliations:** 1 Department of Hygiene and Preventive Medicine (Zoonoses), Faculty of Veterinary Medicine, Kafrelsheikh University, Kafrelsheikh, Egypt; 2 Department of Animal Medicine, Faculty of Veterinary Medicine, Kafrelsheikh University, Kafrelsheikh, Egypt; 3 Department of Biotechnology, College of Science,, Taif University, P.O. Box 11099, Taif, 21944 Saudi Arabia; 4 Food Research Institute, Agriculture Research Center, Giza, Egypt; 5 Department of Bacteriology, Mycology and Immunology, Faculty of Veterinary Medicine, Kafrelsheikh University, Kafrelsheikh, Egypt; 6 Department of Biology, College of Science, Taibah University, Madina, Saudi Arabia; 7 Department of Microbiology and Immunology, Faculty of Medicine, Tanta University, Tanta, Egypt; 8 Department of Poultry and Rabbit Diseases, Faculty of Veterinary Medicine, Kafrelsheikh University, Kafrelsheikh, Egypt; 9 Department of Physiology, Faculty of Veterinary Medicine, Kafrelsheikh University, Kafrelsheikh, Egypt; 10 Department of Bacteriology, Mycology, and Immunology, Faculty of Veterinary Medicine, University of Sadat City, Sadat, Egypt

**Keywords:** Shiga toxin-producing *Escherichia coli*, Carbapenemase genes, Extended-spectrum β-lactamase genes, Multidrug-resistant, Cattle, Public health risk

## Abstract

Shiga toxin-producing *Escherichia coli* (STEC) is a pathotype of *E. coli* that causes enteric and systemic diseases ranging from diarrhoea to severe hemorrhagic colitis (HC) and hemolytic uremic syndrome (HUS). The emergence of multidrug-resistant (MDR) STEC from cattle sources has increased public health risk and limited treatment options. The prevalence of STEC was investigated in 200 raw food samples (milk and beef samples) and 200 diarrheic samples (cattle and human samples) in a matched region. The presence of *stx* genes (*stx1* and *stx2*), carbapenemase-encoding genes (*bla*_VIM_, *bla*_NDM-1_, and *bla*_IMP_), and extended-spectrum β-lactamase (ESBL)-encoding genes (*bla*_TEM_ group, *bla*_CTX-M1_ group, and *bla*_OXA-1_ group) was screened by polymerase chain reaction (PCR). Antibiogram and Enterobacterial repetitive intergenic consensus (ERIC)-PCR were also conducted. STEC isolates were identified in 6.5% (13/200) of food samples [6% (6/100) of milk and 7% (7/100) of beef samples] and in 11% (22/200) of diarrheic cases [12% (12/100) of cattle and 10% (10/100) of human samples]. We found that O26 (4.5%, 18/400) and O111 (1.5%, 6/400) were the most prevalent STEC serovars and were found more commonly in diarrheic samples. STEC strains with both *stx* genes, *stx2* only, and *stx1* only genotypes were present in 62.9% (22/35), 20% (7/35), and 17.1% (6/35) of isolates, respectively. Carbapenemase-producing STEC (CP STEC) isolates were found in 1.8% (7/400) of samples [0.5% (1/200) of foods and 3% (6/200) of diarrheic cases]. The *bla*_VIM_ gene was detected in all CP STEC isolates, and one human isolate carried the *bla*_NDM-1_ gene. ESBL-producing STEC strains were detected in 4.3% (17/400) of samples [1.5% (3/200) of food samples and 7% (14/200) of diarrheic cases]. The *bla*_TEM_, *bla*_CTX-M1_, and *bla*_OXA-1_ genes were detected in 42.9% (15/35), 28.6% (10/35), and 2.9% (1/35) of STEC isolates, respectively. Approximately half (51.4%, 18/35) of STEC isolates were MDR STEC; all CP STEC and ESBL-producing STEC were also MDR STEC. The highest antimicrobial resistance rates were found against nalidixic acid (51.4%) and ampicillin (48.6%), whereas the lowest rates were reported against gentamicin (5.7%) and ciprofloxacin (11.4%). MDR STEC strains were 5.3 times more likely to be found in diarrheic cases than in foods (*P* = 0.009, 95% CI 1.5–18.7). ERIC-PCR was used for genotyping STEC isolates into 27 different ERIC-types (ETs) with a discrimination index of 0.979. Five ETs showed clusters of 2–4 identical isolates that shared the same virulence and antibiotic resistance genetic profile. Human isolates matched food isolates in two of these ET clusters (the O26 CP STEC cluster and the O111 STEC cluster), highlighting the potential cross-species zoonotic transmission of these pathogens and/or their genes in the study region. This is the first detection of CP STEC in milk and diarrheic cattle in Egypt.

## Introduction

Shiga toxin-producing *Escherichia coli* (STEC) strains are among the most important causes of foodborne illness worldwide [1]. Human infection with these pathogens may result in clinical illness ranging from self-limiting diarrhoea to life-threatening hemolytic uremic syndrome (HUS) [2]. Cattle are attributed to most zoonotic human STEC cases worldwide [1–3]. These animals are the main reservoir of O157 STEC and some important non-O157 STEC such as O26, O111, O113, and O103 [1–4]. *E. coli* O157:H7 is the predominant STEC serotype associated with human disease and the leading cause of HUS [1, 3]. However, O26, O111, and O103 are also involved in severe human diseases occurring worldwide [1, 3, 5, 6]. Most STEC serotypes cause no illness in cattle; however, some serotypes, including O157, O26, O5, and O113, cause diarrhoea, particularly in young calves [7]. Cattle may transmit STEC infections to humans through the consumption of raw or inadequately cooked beef (or products), raw or poorly pasteurized milk (or products), vegetables contaminated by their feces, and via direct occupational contact with live carrier animals or their raw products [1, 2, 8].

Antimicrobial-resistant pathogens are one of the most threatening public health problems and are predicted to cause the death of 10 million people annually by 2050 [9]. STEC isolates that carry extended-spectrum β-lactamase (ESBL)-producing genes were reported in humans and cattle sources worldwide [10–13]. These ESBL-producing genes confer resistance to a wide range of β-lactams, which are the most commonly used antibiotics in clinical and veterinary practices. Additionally, carbapenemase reports (Metallo-β-lactamase)-producing clinical *E. coli* isolates in humans are increasing worldwide [14, 15]. This is a more pressing public health concern since carbapenemases, which hydrolyze carbapenems, have been used as a last resort against multidrug-resistant (MDR) pathogens. This is critical for STEC because meropenem (MEM, a carbapenem) is recommended to treat early-stage STEC human infections to prevent HUS and subsequent kidney damage [9]. The emergence of carbapenemase-producing STEC (CP STEC) indicates that these isolates could progress to life-threatening diseases with limited treatment options. Carbapenems are not used in veterinary practices; however, recent reports have identified carbapenemase-carrying *E. coli* in clinical cattle cases [16, 17]. This emergence of CR in cattle isolates may be attributed to either natural selection in the environment or to a human source through the cross-species transmission of these pathogens or their genetic determinants [14, 17]. The potential zoonotic transmission of these pathogens warrants monitoring for CP-*E. coli* in the cattle food chain and other clinical sources. Egypt is part of the Middle East, and this region has the highest annual incidence rates of human STEC cases (152.6/10^5^ people/year; 160 HUS cases) compared with other areas worldwide [8].

Furthermore, STEC isolates were recovered from cattle sources, including clinical cases and foods in Egypt [13, 18]. Some of these isolates showed a variable degree of antibiotic resistance; however, there are no data on CP STEC isolates obtained from cattle sources. Therefore, this study aimed to (1) investigate the occurrence of β-lactam-resistant (including carbapenems) STEC in raw foods of cattle origin (raw beef and milk), diarrheic cattle cases, and diarrheic human cases sharing the same geographical region in Egypt; (2) detect the molecular determinants of their resistance; and (3) define the genetic relatedness or diversity of the isolates for evidence of potential inter- and cross-species (zoonotic) transmission in the study region.

## Materials and methods

### Sampling

Samples were collected from various foods, diarrheic cattle, and diarrheic humans in several Kafrelsheikh governorate districts in the mid-Delta region of Egypt during the period between March and August 2016. A total of 400 samples were collected, including (1) 200 food samples (100 raw beef and 100 raw milk samples) collected from retail markets; approximately 250–500 (mL or g) were purchased of each food sample; (2) rectal swab samples collected from 100 diarrheic cattle cases (two swabs per case) admitted to private veterinary clinics in different regions of the Kafrelsheikh governorate; and (3) swab samples collected from the fresh stool of 100 diarrheic humans (two swabs per case) admitted to the Kafrelsheikh general hospital and six private laboratories in different districts. Diarrheic cases were defined as those with more than three loose stools or feces within 24 h. All swab samples were collected from diarrheic cases (humans or cattle) before initiating antibiotic therapy. The samples were shipped while chilled in an icebox to the laboratory for further analysis.

### *Escherichia coli* isolation

After arriving to the lab, the collected samples were enriched in Tryptone Soy broth (TSB; Oxoid, Hampshire, UK) and TSB with 20 mg/L Novobiocin (mTSB for O157; Oxoid, Hampshire, UK). The rectal/stool swabs were enriched in 10 ml of TSB/mTSB broth. The meat samples were homogenized in TSB/mTSB broth (25 g/225 mL broth) for 2 min at 230 rpm using a Stomacher^®^ 400 Circulator (Seward, Worthing, UK). Twenty-five milliliters of each milk sample was enriched in 225 ml of TSB/mTSB broth. The inoculated broths of all samples were incubated at 37 °C for 6–18 h. Loopfuls from the enrichment tubes were spread on MacConkey agar, Eosin Methylene Blue (EMB agar), and Sorbitol MacConkey agar with Cefixime-Tellurite supplement (CT-SMAC for O157). All media were supplied by Oxoid (Hampshire, UK). The inoculated plates were incubated at 37 °C for 18–24 h. Suspected *E. coli* colonies were confirmed biochemically using API-20E (bioMérieux, Marcy-l'Etoile, France).

### Molecular identification and serotyping of STEC isolates

The STEC isolates were identified by the molecular detection of the *stx1* and *stx2* genes, as described before [19]. In brief, bacterial DNA was extracted from the overnight incubated TSB culture using a QIAamp DNA Mini Kit (Qiagen, Hilden, Germany) according to the manufacturer’s instructions. Duplex PCR was conducted to detect the *stx1* and *stx2* genes [20] using a mixture consisting of 25 μL of EmeraldAmp MAX PCR master mix (Takara Bio, Kusatsu, Japan), one μL (20 pmol) of each primer, five μL of DNA template (~ 100 ng), and water to reach a final reaction volume of 50 μL. The PCR cycling started with an initial denaturation at 94 °C for 7 min; 35 cycles of 94 °C for 1 min, 60 °C for 1 min, and 72 °C for 2 min; and a final extension at 72 °C for 10 min. The primer sequences (Metabion, Steinkirchen, Germany) are shown in Table 1. The *E. coli* O157:H7 Sakai (positive for the *stx1* and *stx2* genes) and *E. coli* ATCC 25922 (negative for the *stx* genes) reference strains were used controls. The PCR reaction was run by the Applied Biosystem 2720 thermal cycler (Applied Biosystem, Foster City, CA, USA). The PCR products were electrophoresed in 1.5% agarose gel containing 0.5 µg/ml ethidium bromide. The gel was visualized using AlphaImager™ Gel Imaging System (Alpha Innotech, San Leandro, CA, USA).

**Table 1:** Primers used in this study and their annealing temperature

Category	Target gene	Primers sequences (5′–3′)	PCR type	Amplified segment (bp)	Annealing temperature (˚C)
Stx	*stx1*	F: ACACTGGATGATCTCAGTGG	Duplex	614	60
		R: CTGAATCCCCCTCCATTATG			
	*stx2*	F: CCATGACAACGGACAGCAGTT		779	
		R: CCTGTCAACTGAGCAGCACTTTG			
MBLs	*bla*_IMP_	F: CATGGTTTGGTGGTTCTTGT	Uniplex	488	55
		R: ATAATTTGGCGGACTTTGGC			
	*bla*_VIM_	F: AGTGGTGAGTATCCGACAG		261	52
		R: ATGAAAGTGCGTGGAGAC			
	*bla*_NDM-1_	F: GGCGGAATGGCTCATCACGA		287	58
		R: CGCAACACAGCCTGACTTTC			
ESBLs	*bla*_OXA-1_ group	F: GGCACCAGATTCAACTTTCAAG	Multiplex	564	61
		R: GACCCCAAGTTTCCTGTAAGTG			
	*bla*_TEM_ group	F: CATTTCCGTGTCGCCCTTATTC		800	
		R: CGTTCATCCATAGTTGCCTGAC			
	*bla*_CTX-M-1_ group	F: TTAGGAAGTGTGCCGCTGTA		655	
		R: CGGTTTTATCCCCCACAAC			

Stx: Shiga like toxin producing genes; MBLs: metallo-β-lactamase (carbapenemase)-producing genes; ESBLs: extended-spectrum β-lactamase producing genes

According to the manufacturer's instructions, confirmed STEC isolates were serotyped using diagnostic *E. coli* O- and H-antisera sets (Denka Seiken Co., Tokyo, Japan).

### Molecular detection of β-lactamase-encoding genes

Metallo-β-lactamase (carbapenemase)-producing genes were detected using a uniplex PCR reaction for the genes *bla*_VIM_ [21], *bla*_IMP_ [22], and *bla*_NDM-1_ [23]. The mixture for each PCR reaction (25 µL) contained 12.5 µL of EmeraldAmp Max PCR Master Mix, 1 µL of each primer (20 pmol), 5 µL of DNA template, and 5.5 µL of water. The following positive control strains were used: *E. coli* NCTC 13476 (positive for *bla*_IMP_), *E. coli* ATCC BAA-2469 (positive for *bla*_NDM-1_), and *K. pneumoniae* NCTC 13439 (positive for *bla*_VIM-1_). Additionally, the isolates were screened for ESBL-encoding genes: the *bla*_TEM_ group gene (encodes for TEM; class A β-lactamases), the *bla*_CTX-M-1_ group gene (encodes for CTX-M; class A β-lactamases)*,* and the *bla*_OXA-1_ group gene (encodes for OXA; class D β-Lactamases). A multiplex PCR was utilized according to [24]. The reaction mixture was similar to that used to detect *stx* genes except for the primers (listed in Table 1). The following PCR cycling conditions were used for all reactions: one cycle at 94 °C for 7 min; 35 cycles of 95 °C for 30 s, annealing temperature per each gene (Table 1) for 40 s, and 72 °C for 1 min; and a final extension at 72 °C for 10 min. The reference strains (*E. coli* ATCC 35218 and *E. coli* NCTC 13353) were used as the positive controls for the *bla*_TEM_ and the *bla*_CTX-M-1_ group genes. For the *bla*_OXA-1_ group gene, an *E. coli* isolate harboring the *bla*_OXA-1_ gene that was kindly provided by the Central Laboratory of Faculty of Veterinary Medicine, Kafrelsheikh University, Egypt, was used as the control. The *E. coli* ATCC 25922 reference strain was used as a negative control for all PCR tests.

### Assessment of phenotypic antibiotic resistance

According to the guidelines of the Clinical and Laboratory Standards Institute, the Kirby–Bauer disk diffusion technique was used to perform the antibiotic sensitivity tests [25]. Pure colonies were incubated in Mueller–Hinton broth (Oxoid, Hampshire, U.K.) at 37 °C for 6 h. Each broth culture was diluted with sterile water until reaching a concentration of 0.5 McFarland standard, and then 100 µL of the dilution was spread on Mueller–Hinton agar (MHA, Oxoid, Hampshire, UK). Antibiotic discs (Oxoid, Hampshire, U.K.) were distributed onto the agar surface with a 30 mm distance from center to center. The following antibiotic discs were used: imipenem (IMP, 10 µg), meropenem (MEM, 10 µg), ampicillin (AMP, 10 µg), cephazolin (30 µg), ceftazidime (CAZ, 30 µg), cefotaxime (CTX, 30 µg), nalidixic acid (NA, 30 µg), ciprofloxacin (CIP, 5 µg), streptomycin (S, 10 µg), kanamycin (K, 30 µg), gentamicin (CN, 10 µg), tetracycline (TE, 30 µg), chloramphenicol (C, 30 µg), and sulfamethoxazole/trimethoprim (SXT, 25 µg). All plates were incubated at 37 °C for 18–24 h, and the inhibition zone diameters were interpreted according to the CLSI guidelines (2016). Isolates that showed phenotypic resistance to CAZ or CTX were further tested for the production of ESBLs by the double-disk synergy test as previously described [26]. Briefly, each isolate was inoculated on the MHA plate, and then an amoxicillin/clavulanic acid disk (AMC, 20/10 μg) was placed 25 mm from the CAZ (30 µg) and CTX (30 µg) disks. After incubation, the increase in the CAZ inhibition zone or CTX disks toward the AMC disk (keyhole shape) was recorded as positive ESBL production. Carbapenem-resistant isolates were tested for carbapenem production with the modified Hodge test [27]. In brief, the tenth dilution of the *E. coli* ATCC 25922 reference strain (0.5 McFarland-equivalent concentration) was inoculated on an MHA plate, and then a MEM disk (10 µg) was placed in the center. Then, the isolates (three per plate) were streaked in a line from the MEM disk to the plate edge, and the plate was incubated overnight. Positive results were considered when *E. coli* ATCC 25922 increased around the test organism’s growth streak within the disk inhibition zone (clover leaf-like indentation).

For quality control, the following reference strains were used for each of antibiotic sensitivity, ESBL production, and carbapenemase production tests: *E. coli* ATCC BAA-2469 (positive control for carbapenemase)*, E. coli* NCTC 13353 (positive control for ESBL), and *E. coli* ATCC 25922 (negative control).

### Genotyping of STEC isolates using Enterobacterial repetitive intergenic consensus (ERIC)-PCR

Genotyping with ERIC-PCR was conducted as previously described [28] using the following primers: ERIC1R: 5′ATGTAAGCTCCTGGGGATTCAC3′ and ERIC2: 5′AAGTAAGTGACTGGGGTGAGCG3′. The reaction mixture was composed of 12.5 µL of EmeraldAmp Max PCR Master Mix, 3 µL of each primer (60 pmol), 5 µL of the DNA template (100 ng), and water to reach a total volume of 25 µL. The following cycling conditions were applied: 1 cycle at 95 °C for 7 min; 35 cycles of 94 °C for 30 s, 52 °C for 1 min, and 65 °C for 5 min; and a final extension at 65 °C for 15 min. The PCR products were electrophoresed and photographed, as mentioned before. The ERIC-PCR band patterns were analyzed by GelJ software v.2.0 [29]. The comparison between ERIC-PCR profiles was conducted using the Dice coefficient, and a dendrogram was constructed using the unweighted pair group method with arithmetic mean. Simpson's discrimination index for ERIC genotyping was estimated as previously described [30].

### Statistical analysis

The odds ratios and potential associations between phenotypic or genetic antibiotic resistance profiles and source (diarrheic cases versus food) of the STEC isolates were assessed using a univariate logistic regression model. The analysis was conducted using SPSS v19 (IBM, Armonk, NY, USA), and significance was recorded at *P* ≤ 0.05.

## Results

STEC were detected in 6.5% (13/200) and 11% (22/200) of foods and diarrheic cases, respectively. STEC prevalence rates of 6% (6/100), 7% (7/100), 10% (10/100), and 12% (12/100) were reported in individual milk, beef, diarrheic human, and diarrheic cattle samples, respectively (Table 2). Six STEC serovars were detected; O26:H11 (4.5%, 18/400) was the most prevalent serovar, followed by O111:H2 (1.5%, 6/400). The O26:H11 strains were detected in all sources, and the highest rates were found in diarrheic cattle (9%, 9/100) and human (4%, 4/100) samples. The O111:H2 strains were only detected in beef (2%, 2/100) and human (4%, 4/100) samples (Table 2).

**Table 2:** Frequency distribution of STEC serovars and drug resistance traits in samples collected from cattle and humans in this study

Serovar	Foods of cattle origin			Diarrheic cases			Total N = 400
	Milk N = 100	Beef N = 100	Subtotal N = 200	Cattle N = 100	Humans N = 100	Subtotal N = 200	
O26:H11	3 (3)	2 (2)	5 (2.5)	9 (9)	4 (4)	13 (6.5)	18 (4.5)
O111:H2	0 (0)	2 (2)	2 (1)	0 (0)	4 (4)	4 (2)	6 (1.5)
O91:H21	2 (2)	0 (0)	2 (1)	1 (1)	1 (1)	2 (1)	4 (1)
O128:H2	0 (0)	3 (3)	3 (1.5)	1 (1)	0 (0)	1 (0.5)	4 (1)
O103:H2	1 (1)	0 (0)	1 (0.5)	0 (0)	1 (1)	1 (0.5)	2 (0.5)
O113:H4	0 (0)	0 (0)	0 (0)	1 (1)	0 (0)	1 (0.5)	1 (0.3)
Total STEC	6 (6)	7 (7)	13 (6.5)	12 (12)	10 (10)	22 (11)	35 (8.8)
CR-STEC	1 (1)	0 (0)	1 (0.5)	2 (2)	4 (4)	6 (3)	7 (1.8)
ESBL-STEC	2 (2)	1 (1)	3 (1.5)	7 (7)	7 (7)	14 (7)	17 (4.3)
MDR-STEC	2 (2)	1 (1)	3 (1.5)	8 (8)	7 (7)	15 (7.5)	18 (4.5)

STEC: Shiga toxin-producing *Escherichia coli*; CR-STEC: carbapenemase-producing STEC; ESBL-STEC: extended-spectrum β-lactamase producing STEC; MDR-STEC: multidrug resistant STEC; brackets: percent: N: number of samples

**Fig. 1:**
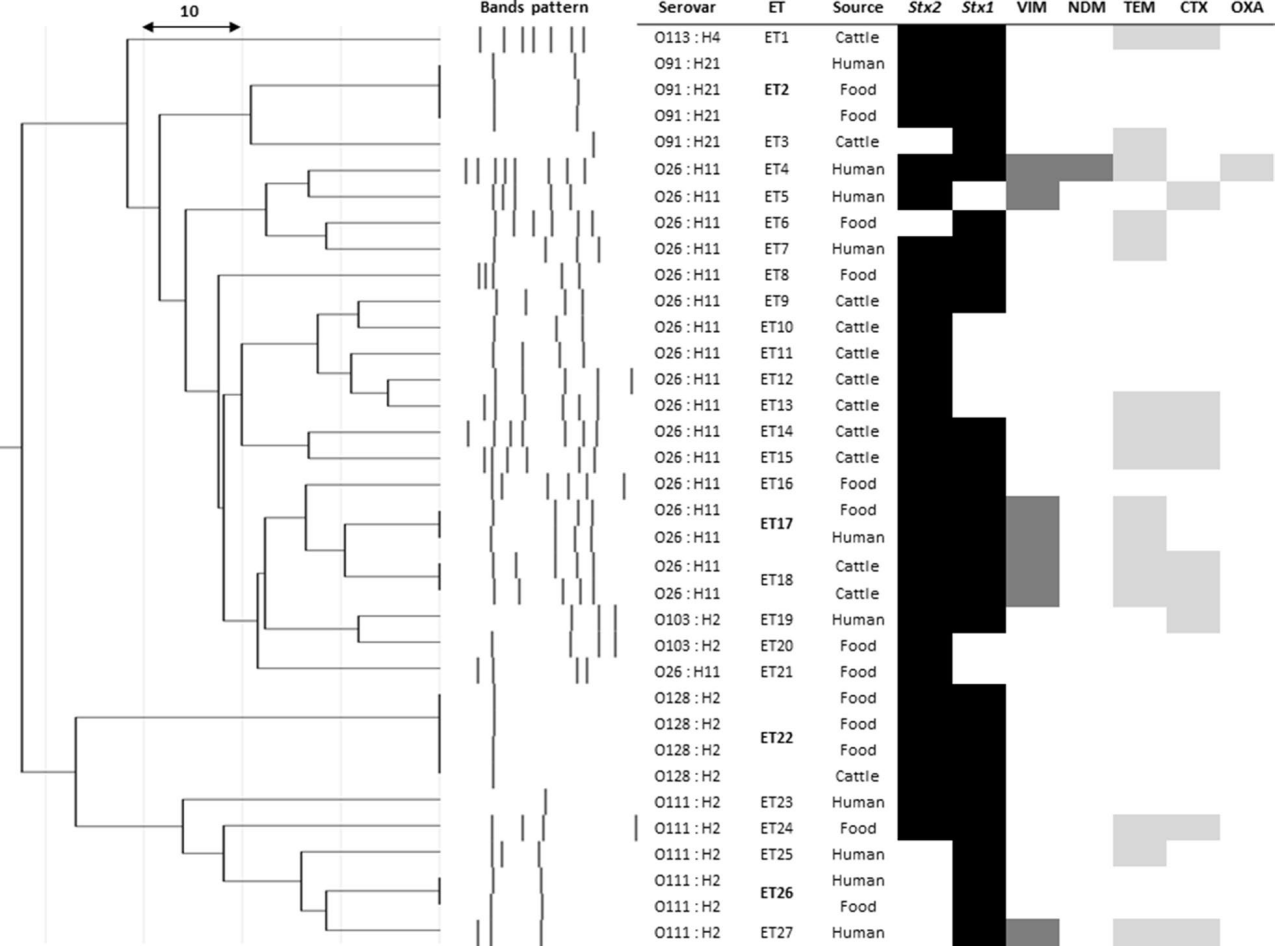
Enterobacterial repetitive intergenic consensus (ERIC)-PCR genotyping and virulence-antibiotic resistance genes profiles of STEC isolates recovered from food of cattle origin, diarrheic cattle, and diarrheic humans in this study. ET: ERIC genotypes. Black shadow: virulence genes. Dark grey shadow: CR genes. Light grey shadow: ESBL genes. Bold ET: clusters of identical isolates

The *stx1* and *stx2* genes were detected in 82.9% (29/35) and 80% (28/35) STEC isolates, respectively. Most STEC isolates harbored both *stx* genes (62.9%, 22/35); the remaining isolates carried either only the *stx2* gene (20%, 7/35) or only the *stx1* gene (17.1%, 6/35). The genotype containing both *stx* genes predominated in all STEC sources: 69.2% (9/13) in foods, 58.3% (7/12) in cattle, and 60% (6/10) in humans. Furthermore, the highest rate of the *stx2*-only genotype was detected in clinical cattle isolates (33.3%, 4/12). For the O26:H11 isolates, the both *stx* genes, the *stx2* gene only, and the *stx1* gene only genotypes were recorded in 61.1% (11/18), 33.3% (6/18), and 5.6% (1/18) of isolates, respectively (Fig. 1). Most O26:H11 isolates carrying the *stx2* gene were detected in diarrheic cattle isolates (4/6 isolates, Fig. 1). The *stx1* gene only (66.7%, 4/6) and both *stx* genes (33.3%, 2/6) were the most prevalent genotypes for the O111:H2 serovar (Fig. 1). The serovar O157 was not detected in any of the examined samples.

CP STEC isolates that harbored at least one of the MBL genes were found in 1.8% of the examined samples, including 0.5% (1/200) of the food samples and 3% (6/200) of the diarrheic cases (Table 2). The *bla*_VIM_ was the most prevalent MBL gene, and it was detected in 20% (7/35) of STEC isolates. One isolate carried the *bla*_NDM-1_ gene with *bla*_VIM_ (2.9%); however, the *bla*_IMP_ gene was not detected in any isolates (Table 2). There was no significant association between the acquisition of CR genes and the STEC isolates' source; however, higher odds ratios were reported for diarrheic isolates (OR 6.2, *P* = 0.09, 95% CI 0.7–51.6).

The ESBL-producing STEC isolates were detected in 4.3% of the samples, including 1.5% of the food samples and 7% of the diarrheic cases (Table 2). Approximately half of the isolates carried ESBL genes (48.6%, 17/35): 42.9% (15/35) carried *bla*_TEM_ group genes, 28.6% (10/35) carried *bla*_CTX-M1_ group genes, and 2.9% (1/35) carried *bla*_OXA-1_ group genes (Table 3). Half (50%, 6/12) of the cattle clinical isolates carried both *bla*_TEM_ and blaCTX-M1 group genes, whereas only one human isolate carried both the *bla*_TEM_ group and *bla*_OXA-1_ group genes (Table 3). The ESBL-producing STEC isolates were 4.9 times more likely to be in clinical samples than in food samples (*P* = 0.006, 95% CI 1.4–17.5).

**Table 3:** Antibiotic resistance phenotypes and their associated resistance genes among cattle, humans and food STEC isolates in this study

Ser	Source	No	Carbapenems resistance				β-Lactams resistance							Other antibiotics resistance								MARi
			Phenotype		Genotype		Phenotype				Genotype			Phenotype								
			IMP	MEM	*bla*_VIM_	*bla*_NDM_	AMP	KZ	CTX	CAZ	*bla*_TEM_	*bla*_CTX_	*bla*_OXA_	NA	CIP	S	K	CN	TE	C	SXT	
O26	Cattle	2	+	+	●		+	+	+	+	●	●		+		+	+		+	+	+	0.86
	Humans	1	+	+	●	●	+	+	+	+	●		●	+	+	+	+		+		+	0.86
	Humans	1	+	+	●		+	+	+	+		●		+		+	+	+		+		0.79
	Cattle	1					+	+	+	+	●	●		+	+	+			+	+		0.64
	Milk	1					+	+			●			+		+	+	+	+		+	0.57
	Cattle	1					+	+	+	+	●	●		+	+	+			+			0.57
	Cattle	1					+	+	+	+	●	●		+		+	+		+			0.57
	Milk	1	+	+	●		+	+			●			+		+	+				+	0.57
	Humans	1																				
	Humans	1					+	+			●					+			+	+		0.36
	Cattle	1														+			+	+		0.21
	Cattle	1												+								0.07
O111	Humans	1	+	+	●		+	+	+	+	●	●		+	+				+			0.64
	Meat	1					+	+	+		●	●		+		+	+		+		+	0.57
	Humans	1					+				●			+		+			+		+	0.36
O91	Cattle	1					+				●			+					+		+	0.29
O103	Humans	1					+	+	+	+		●		+					+			0.43
	Milk	1												+		+						0.14
O113	Cattle	1					+	+	+		●	●		+		+			+	+	+	0.57
Total	No	20	7	7	7	1	17	15	11	9	15	10	1	18	4	16	9	2	15	7	10	
	%	57.1	20	20	20	2.9	48.6	42.9	31.4	25.7	42.9	28.6	2.9	51.4	11.4	45.7	25.7	5.7	42.9	20	28.6	

Isolates with no detected antibiotic resistance: O26 [2 cattle and 3 food isolates]; O111 [2 humans and 1 food isolates]; O91 [2 food and 1 humans isolates]; O128 [3 food and 1 cattle isolates]; Ser.: Serovar; No.: number of isolates; IMP: imipenem (IMP 10 µg); MEM: meropenem (MEM 10 µg); AMP: ampicillin (AMP 10 µg); KZ: cephazolin (KZ 30 µg); CAZ: ceftazidime (CAZ 30 µg); CTX: cefotaxime (CTX 30 µg); NA: Nalidixic acid (NA 30 µg); CIP: ciprofloxacin (CIP 5 µg); S: streptomycin (S 10 µg); K: kanamycin (K 30 µg); CN: gentamicin (CN 10 µg); TE: tetracycline (TE 30 µg); C: chloramphenicol (C 30 µg); SXT: sulfamethoxazole/trimethoprim (SXT 25 µg); MARi: multiple antibiotic resistance index; Plus ( +): positive phenotypic resistance; Black dots (**●**): positive detection of antibiotic resistance gene

Approximately two-thirds (57.1%, 20/35) of the examined STEC isolates were phenotypically resistant to at least one antibiotic, and 18 (51.4%) isolates were MDR to three or more classes of antibiotics, including eight cattle (22.9%), seven human (20%), and three food (8.6%) isolates (Table 3). MDR STEC strains were 5.3 times more likely to be detected in diarrheic cases than in foods (*P* = 0.009, 95% CI 1.5–18.7). Seven (20%) isolates were resistant to the tested carbapenems (IMP and MEM). The highest resistant rates were reported for NA (51.4%), AMP (48.6%), and S (45.7%), while the highest sensitivity rates were reported for CN (5.7%), and CIP (11.4%).

There was a concordance between the acquisition of carbapenemase- and β-lactamase-producing genes and the expression of phenotypic carbapenem and β-lactam resistance in all studied STEC isolates, respectively (Table 3). Interestingly, all carbapenem-resistant isolates were also resistant to β-lactams and harbored one or more other BL genes.

The ERIC-PCR based genotyping analysis of the STEC isolates from clinical cases (cattle and humans) and food products (milk and beef) is shown in Fig. 1. The ERIC band patterns ranged from 1 to 8 bands with a size range from 100 to 2000 bp. The dendrogram map classified the STEC isolates into 27 different ERIC genotypes (ETs) with a discrimination index of 0.979. The isolates that belonged to the same serotype were clustered together (Fig. 1). The isolates belonging to serovars O26 (18 isolates displaying 16 ETs) and O111 showed high genetic diversity: 18 isolates displayed 16 ETs and 6 isolates displayed 5 ETs, respectively. By contrast, more relatedness was exhibited by the isolates of serovars O91 and O128: four isolates showed two ETs and four isolates showed one ET, respectively. Five ETs showed clusters of two to four identical isolates per ET (Fig. 1). These ET clusters either belonged to the same source (E18, diseased cattle) or were from different sources, such as diseased humans and foods (ET2, ET17, and ET26) and diseased cattle and foods (ET22). The isolates within the same ET shared identical virulence and antibiotic resistance genetic profiles (Fig. 1).

## Discussion

This study investigated the prevalence and antibiotic resistance traits of STEC in the food of cattle origin (milk and meat) and diarrheic cases (cattle and humans). STEC was detected in 6.5%, 6%, and 7% of all food, milk, and meat samples, respectively. These findings were higher than those in previous reports (1.9–4.1%) in the USA [31] but lower than reports (10.7–29.7%) from other countries [32–34]. STEC isolates were found in 12% of diarrheic cattle samples; higher rates (18.7–53.2%) were reported from another location in Egypt [18] and elsewhere [31, 35]. The prevalence rate in humans was 10%, which was higher than that in other reports (0.7–6.4%) in Africa [36], Europe [37], and Asia [35]. Most (60–69.2%) of the food and human isolates displayed the genotype with both *stx* genes. Similarly, the same genotype predominated in human isolates from Canada [38] and Europe [37]. However, our findings differed from reports of food isolates [32, 34] and human isolates [36] found elsewhere. By contrast, the *stx2*-only genotype prevailed in diarrheic cattle isolates in this study, which agreed with reports from Canada [38], Argentina [10], and Egypt [18].

The serovars O26:H11 (4.5%) and O111:H2 (1.5%) were the most commonly detected STEC among the examined samples. Similarly, these serovars were detected in the food of cattle origin, live cattle, and diseased humans worldwide [4, 10, 32, 34, 39]. The O26:H11 serovar was reported to be the most frequently recorded non-O157 STEC responsible for human disease worldwide [10]. The most prevalent genotypes for the O26:H11 serovar were both *stx* genes (61.1%) and *stx2* only (33.3%). The STEC O26 genotype with both *stx* genes has caused several human cases of bloody diarrhoea and HUS in the USA and Europe [1, 5, 40]. The STEC O26 genotype with *stx2* only emerged in Europe in the mid-1990s and continues to be the most common non-O157 STEC etiology of HUS worldwide [1, 40]. Interestingly, most of the STEC O26 isolates with the *stx2* genotype were recovered from clinical cattle cases in this study, which highlights the potential zoonotic risk of this serovar. The O111:H2 serovar displayed two genotypes: *stx1* only (66.7%) and both *stx* (33.3%). STEC O111 strains (*stx1* or both *stx* genes) were the leading cause of HUS cases in the USA from 1983 to 2002 [5]. The serovar O157 was not found in any of the examined samples. There is increasing global evidence over recent years of the increased prevalence of non-O157 STEC isolates among cattle and human samples [4, 10, 39], which agrees with our findings.

Differences in STEC prevalence rates, genotypes, and serogroups between this study and previous studies may be attributed to differences in the geographical distribution of STEC strains, the sampling strategy, or the methodology.

Seven CP STEC isolates were found in 1.8% of our examined samples, including in one milk (1%), two diarrheic cattle (2%), and four diarrheic human (4%) samples. These isolates carried the *bla*_VIM_ gene, and one human isolate harbored both *bla*_VIM_ and *bla*_NDM-1_ genes; all of these isolates were also phenotypically resistant to IMP and MEM. Human CR-*E. coli* isolates harboring the *bla*_VIM_ or *bla*_NDM_ genes were identified in clinical isolates from Egypt [41] and other countries [15–17], which agreed with the study findings. Recent reports have shown the emergence of CR-*E. coli* isolates recovered from diarrheic cattle (carrying the *bla*_VIM_ gene) and mastitic milk (carrying the *bla*_NDM_ gene) in India. This agrees with the current study findings. By contrast, STEC isolates recovered from milk, and diarrheic cattle in previous studies from Egypt showed a complete sensitivity for carbapenems [13, 18]. This is the first detection of CP STEC in milk and diarrheic cattle in Egypt. The emergence of CP STEC is an alarming threat to public health. MEM is recommended for treating STEC human cases to lower severe outcomes such as kidney damage [9]; thus, CP STEC may be life-threatening and has reduced therapeutic options. Unlike humans, carbapenems are not used in veterinary practice in Egypt and several other countries [14], so the acquisition of CP genes by cattle isolates may have originated from the environment, cross-species transmission of human CP isolates, and/or the transfer of CP genes via mobile genetic elements as plasmids from other CP-gut pathogens [14, 17]. Despite growing records of non-human sources of CR-*E. coli* worldwide, the role of cattle in the spread of CP STEC to humans or the environment is highly underestimated. This study provides additional evidence of the potential role of cattle and foods of cattle origin as CP STEC sources in the study area, which presents an emerging threat to public health.

Half of the STEC isolates carried ESBL-encoding genes (48.6%); the respective detection rates of the examined *bla*_TEM_, *bla*_CTX-M1_ group, and *bla*_OXA-1_ group genes were 42.9%, 28.6%, and 2.9%. The relative predominance of the *bla*_TEM_ gene in STEC isolates from foods, diseased cattle, and diseased humans has been recorded in several studies worldwide [10, 11, 42]. The *bla*_CTX-M1_ and *bla*_OXA-1_ genes were also recovered at variable rates from the same sources [11, 13, 42]. The *bla*_TEM_ and *bla*_OXA-1_ group genes encode variable narrow-spectrum BLs (NSBL) to ESBLs that confer resistance for penicillin and sometimes cephalosporins. In contrast, the *bla*_CTX-M1_ group gene confers resistance to ESBLs such as third-generation cephalosporins [43]. The genetic profile of all ESBL-producing STEC isolates matched their phenotypic resistance. Interestingly, all CP STEC harbored one or more ESBL genes, which was in agreement with another study in Africa [15]. Additionally, ESBL-producing STEC isolates were five times more likely to be detected in clinical samples than in food samples (*P* = 0.006). These findings suggest the potential acquisition of ESBL genes by selective antibiotic pressure, particularly in clinical isolates (veterinary and humans), usually treated by cephalosporins.

The STEC isolates showed high phenotypic resistance rates to NA (51.4%), AMP (48.6%), S (45.7%), and TE (42.9%). Comparable findings were previously recorded in Egypt [13, 42] and elsewhere [32, 34]. The highest STEC isolates sensitivity rates were reported for CN (5.7%) and CIP (11.4%), which agrees with previous reports in Africa [13, 36]. By contrast, STEC isolates from Asia showed high resistance rates (55.2–100%) to CN [32, 34].

Approximately half (51.4%, 18/35) of the STEC isolates showed the MDR phenotype, which agrees with other reports [13, 34, 42]. However, Kalule et al. [36] reported that none of the detected STEC isolates showed MDR in South Africa. Two-thirds (61.1%, 11/18) of the MDR STEC were from diarrheic cattle and food samples. This finding denotes the emergence of MDR STEC from cattle and their food products in Egypt. In Egypt, antibiotics are misused in veterinary practices. Animals' owners can easily access antibiotics at local pharmaceutical vendors or private pharmacies without a prescription or supervision. This misuse of antibiotics may have contributed to our high recorded MDR STEC rates from animal sources and is a major zoonotic threat to residents in Egypt.

ERIC-PCR genotyping of the 22 clinical and 13 food isolates yielded 27 different ETs. This proved the high genetic diversity that exists between STEC isolates regardless of their source or serotype. Likewise, other studies on pathogenic *E. coli* isolates from clinical cases, and foods showed high genetic heterogeneity [15, 44, 45]. The ERIC band patterns ranged from 1 to 8 bands with a size range from 100 to 2000 bp, comparable with reports from China [45] and Ghana [15]. The STEC isolates of the same serotype were clustered together; however, there was a high genetic difference between strains of some serotypes such as O26 and O111. This agrees with other studies [44, 45] and may indicate the circulation of many different strains of these serotypes in the study area.

Five identical ETs were spotted from either the same source (diseased cattle) or different sources (diseased humans and food; diseased cattle and food); isolates with identical ETs carried matched virulence and antibiotic resistance profiles. The combinations of identical genetic ETs, virulence, and resistance profiles among some of the STEC isolates from the same or different sources highlight the potential inter or intra-species cross-transmission of these pathogens and/or their genes in the study region.

## Conclusions

This work has confirmed a direct role of cattle as a source of CP STEC isolates. It has provided evidence of potential zoonotic transmission of these isolates to humans, representing an emerging public health threat in the study region. ESBL-producing STEC isolates were also recovered from diarrheic cattle and their food products. Taken together, we propose that extended surveillance of the cattle food chain and other clinical sources and mandatory veterinary supervision of antibiotic use for animals are urgently required to minimize the potential zoonotic risks of MDR STEC in Egypt.

## Data Availability

The datasets used in the present study are accessible on reasonable request from the corresponding author.

## References

[1] Organization WH (2019). Shiga toxin-producing *Escherichia coli* (STEC) and food: attribution characterization and monitoring.

[2] Mughini-Gras L, Van Pelt W, Van der Voort M, Heck M, Friesema I, Franz E (2018). Attribution of human infections with Shiga toxin-producing *Escherichia coli* (STEC) to livestock sources and identification of source-specific risk factors, The Netherlands (2010–2014). Zoonoses Public Health.

[3] Martens SL, Klein S, Barnes RA, TrejoSanchez P, Roth CC, Ibey BL (2020). 600-ns pulsed electric fields affect inactivation and antibiotic susceptibilities of *Escherichia coli* and *Lactobacillus acidophilus*. AMB Express.

[4] Jajarmi M, Fooladi AAI, Badouei MA, Ahmadi A (2017). Virulence genes, Shiga toxin subtypes, major O-serogroups, and phylogenetic background of Shiga toxin-producing *Escherichia coli* strains isolated from cattle in Iran. Microb Pathog.

[5] Brooks JT, Sowers EG, Wells JG, Greene KD, Griffin PM, Hoekstra RM (2005). Non-O157 Shiga toxin–producing *Escherichia coli* infections in the United States, 1983–2002. J Infect Dis.

[6] Bielaszewska M, Mellmann A, Bletz S, Zhang W, Köck R, Kossow A (2013). Enterohemorrhagic *Escherichia coli* O26: H11/H−: a new virulent clone emerges in Europe. Clin Infect Dis.

[7] Hornitzky MA, Mercieca K, Bettelheim KA, Djordjevic SP (2005). Bovine feces from animals with gastrointestinal infections are a source of serologically diverse atypical enteropathogenic *Escherichia coli* and Shiga toxin-producing *E. coli* strains that commonly possess intimin. Appl Environ Microbiol.

[8] Majowicz SE, Scallan E, Jones-Bitton A, Sargeant JM, Stapleton J, Angulo FJ (2014). Global incidence of human Shiga toxin-producing *Escherichia coli* infections and deaths: a systematic review and knowledge synthesis. Foodborne Pathog Dis.

[9] Mir RA, Kudva IT (2019). Antibiotic-resistant Shiga toxin-producing *Escherichia coli*: an overview of prevalence and intervention strategies. Zoonoses Public Health.

[10] Krüger A, Lucchesi P, Sanso AM, Etcheverría AI, Bustamante AV, Burgán J (2015). Genetic characterization of Shiga toxin-producing *Escherichia coli* O26: H11 strains isolated from animal, food, and clinical samples. Front Cell Infect Microbiol.

[11] Day M, Doumith M, Jenkins C, Dallman TJ, Hopkins KL, Elson R (2016). Antimicrobial resistance in Shiga toxin-producing *Escherichia coli* serogroups O157 and O26 isolated from human cases of diarrhoeal disease in England, 2015. J Antimicrob Chemother.

[12] Nagel S, Spüler M (2019). World’s fastest brain–computer interface: combining EEG2Code with deep learning. PLoS ONE.

[13] Elafify M, Khalifa HO, Al-Ashmawy M, Elsherbini M, El Latif AA, Okanda T (2020). Prevalence and antimicrobial resistance of Shiga toxin-producing *Escherichia coli* in milk and dairy products in Egypt. J Environ Sci Health Part B.

[14] Köck R, Daniels-Haardt I, Becker K, Mellmann A, Friedrich AW, Mevius D (2018). Carbapenem-resistant Enterobacteriaceae in wildlife, food-producing, and companion animals: a systematic review. Clin Microbiol Infect.

[15] Codjoe FS, Donkor ES (2018). Carbapenem resistance: a review. Med Sci.

[16] Ghatak S, Singha A, Sen A, Guha C, Ahuja A, Bhattacharjee U (2013). Detection of New Delhi metallo-beta-lactamase and extended-spectrum beta-lactamase genes in *Escherichia coli* isolated from mastitic milk samples. Transboundary Emerg Dis.

[17] Murugan MS, Sinha D, Kumar OV, Yadav AK, Pruthvishree B, Vadhana P, et al. Epidemiology of carbapenem-resistant *Escherichia coli* and first report of blaVIM carbapenemases gene in calves from India. Epidemiol. Infect. 2019;147. 10.1017/S0950268819000463 PMC6518490 31063112

[18] Sobhy NM, Yousef SG, Aboubakr HA, Nisar M, Nagaraja KV, Mor SK (2020). Virulence factors and antibiograms of *Escherichia coli* isolated from diarrheic calves of Egyptian cattle and water buffaloes. PLoS ONE.

[19] Fagan PK, Hornitzky MA, Bettelheim KA, Djordjevic SP (1999). Detection of Shiga-like toxin (stx1 andstx2), intimin (eaeA), and enterohemorrhagic *Escherichia coli* (EHEC) hemolysin (EHEC hlyA) genes in animal feces by multiplex PCR. Appl Environ Microbiol.

[20] Gannon V, King RK, Kim JY, Thomas E (1992). Rapid and sensitive method for detection of Shiga-like toxin-producing *Escherichia coli* in ground beef using the polymerase chain reaction. Appl Environ Microbiol.

[21] Tsakris A, Pournaras S, Woodford N, Palepou M-FI, Babini GS, Douboyas J (2000). Outbreak of infections caused by *Pseudomonas aeruginosa* producing VIM-1 carbapenemase in Greece. J Clin Microbiol.

[22] Ruiz M, Marti S, Fernandez-Cuenca F, Pascual A, Vila J (2007). High prevalence of carbapenem-hydrolysing oxacillinases in epidemiologically related and unrelated *Acinetobacter baumannii* clinical isolates in Spain. Clin Microbiol Infect.

[23] Caporaso JG, Kuczynski J, Stombaugh J, Bittinger K, Bushman FD, Costello EK (2010). QIIME allows analysis of high-throughput community sequencing data. Nat Methods.

[24] Ogutu JO, Zhang Q, Huang Y, Yan H, Su L, Gao B (2015). Development of a multiplex PCR system and its application in detection of bla SHV, bla TEM, bla CTX-M-1, bla CTX-M-9 and bla OXA-1 group genes in clinical *Klebsiella pneumoniae* and *Escherichia coli* strains. J Antibiot.

[25] Wayne A. Clinical and Laboratory Standards Institute; CLSI. 2011. Performance standards for antimicrobial susceptibility testing. 20th Informational Supplement. CLSI document.

[26] Drieux L, Brossier F, Sougakoff W, Jarlier V (2008). Phenotypic detection of extended-spectrum β-lactamase production in Enterobacteriaceae: review and bench guide. Clin Microbiol Infect.

[27] Amjad A, Mirza IA, Abbasi S, Farwa U, Malik N, Zia F (2011). Modified Hodge test: a simple and effective test for detection of carbapenemase production. Iran J Microbiol.

[28] Versalovic J, Koeuth T, Lupski R (1991). Distribution of repetitive DNA sequences in eubacteria and application to finerpriting of bacterial enomes. Nucleic Acids Res.

[29] Heras J, Domínguez C, Mata E, Pascual V, Lozano C, Torres C (2015). GelJ—a tool for analyzing DNA fingerprint gel images. BMC Bioinform.

[30] Hunter PR, Gaston MA (1988). Numerical index of the discriminatory ability of typing systems: an application of Simpson's index of diversity. J Clin Microbiol.

[31] Lambertini E, Karns JS, Van Kessel JAS, Cao H, Schukken YH, Wolfgang DR (2015). Dynamics of *Escherichia coli* virulence factors in dairy herds and farm environments in a longitudinal study in the United States. Appl Environ Microbiol.

[32] Momtaz H, Dehkordi FS, Rahimi E, Ezadi H, Arab R (2013). Incidence of Shiga toxin-producing *Escherichia coli* serogroups in ruminant's meat. Meat Sci.

[33] Samadpour M, Kubler M, Buck F, Depavia G, Mazengia E, Stewart J (2002). Prevalence of Shiga toxin-producing *Escherichia coli* in ground beef and cattle feces from King County. Wash. J Food Prot..

[34] Ranjbar R, Dehkordi FS, Shahreza MHS, Rahimi E (2018). Prevalence, identification of virulence factors, O-serogroups and antibiotic resistance properties of Shiga-toxin producing *Escherichia coli* strains isolated from raw milk and traditional dairy products. Antimicrob Resist Infect Control.

[35] Badouei MA, Jajarmi M, Mirsalehian A (2015). Virulence profiling and genetic relatedness of Shiga toxin-producing *Escherichia coli* isolated from humans and ruminants. Comp Immunol Microbiol Infect Dis.

[36] Kalule JB, Keddy KH, Nicol MP (2018). Characterisation of STEC and other diarrheic *E. coli* isolated on CHROMagar™ STEC at a tertiary referral hospital, Cape Town. BMC Microbiol.

[37] Falup-Pecurariu O, Lixandru RI, Cojocaru E, Csutak K, Monescu V, Muhsen K (2019). Shiga toxin producing *Escherichia coli*-associated diarrhea and hemolytic uremic syndrome in young children in Romania. Gut Pathog.

[38] Chandran A, Mazumder A (2013). Prevalence of diarrhea-associated virulence genes and genetic diversity in *Escherichia coli* isolates from fecal material of various animal hosts. Appl Environ Microbiol.

[39] Hussein H, Bollinger L (2005). Prevalence of Shiga toxin-producing *Escherichia coli* in beef. Meat Sci.

[40] Bialaszewski D (2013). Student attitudes regarding Ebooks; a survey with cost savings implications. J Syst Cybern Inform.

[41] El-Kholy AA, Girgis SA, Shetta MA, Abdel-Hamid DH, Elmanakhly AR. Molecular characterization of multidrug-resistant Gram-negative pathogens in three tertiary hospitals in Cairo, Egypt. Eur J Clin Microbiol Infect Dis. 2020:1–6. 10.1007/s10096-020-03812-z PMC7182536 31953591

[42] Ahmed AM, Shimamoto T (2015). Molecular analysis of multidrug resistance in Shiga toxin-producing *Escherichia coli* O157: H7 isolated from meat and dairy products. Int J Food Microbiol.

[43] Kim Y-R, Kim S-I, Lee J-Y, Park Y-J, Lee K-Y, Kang M-W (2005). Nosocomial transmission of CTX-M-15 and OXA-30 β-lactamase-producing *Escherichia coli* in a neurosurgical intensive care unit. Ann Clin Lab Sci.

[44] Prabhu V, Isloor S, Balu M, Suryanarayana V, Rathnamma D. Genotyping by ERIC-PCR of *Escherichia coli* isolated from bovine mastitis cases. 2010.

[45] Zhang S, Wu Q, Zhang J, Zhu X (2016). Occurrence and characterization of enteropathogenic *Escherichia coli* (EPEC) in retail ready-to-eat foods in China. Foodborne Pathog Dis.

